# Supplementary material for: Long-Term Systemic Expression of a Novel PD-1 Blocking Nanobody from an AAV Vector Provides Antitumor Activity without Toxicity
Source: Biomedicines. 2020 Dec 2;8(12):562. doi: 10.3390/biomedicines8120562 (PMC7761623; doi:10.3390/biomedicines8120562)
Supplement: Supplementary file 1 [file biomedicines-08-00562-s001.pdf]

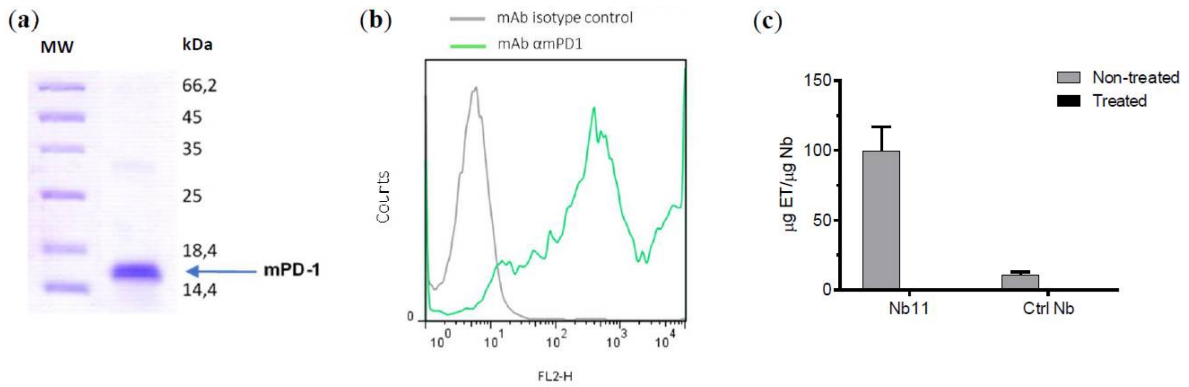

Supplementary figure 1. Quality assessment of recombinant mPD-1, HEK-293T/mPD-1+ cells and recombinant endotoxin-free Nbs. (**a**) Coomassie staining analysis of mPD-1 purified from *E. coli*, used for llama immunization. (**b**) Flow-cytometry analysis of mPD-1 expression on transiently transfected HEK-293T cells used as substrate for bio-panning, using a commercial anti-PD1 mAb (αmPD1). (**c**) Analysis of endotoxin (ET) presence in Nbs expressed from *E. coli*, before and after treatment with ET removal columns. No ET contamination was detected in treated samples.

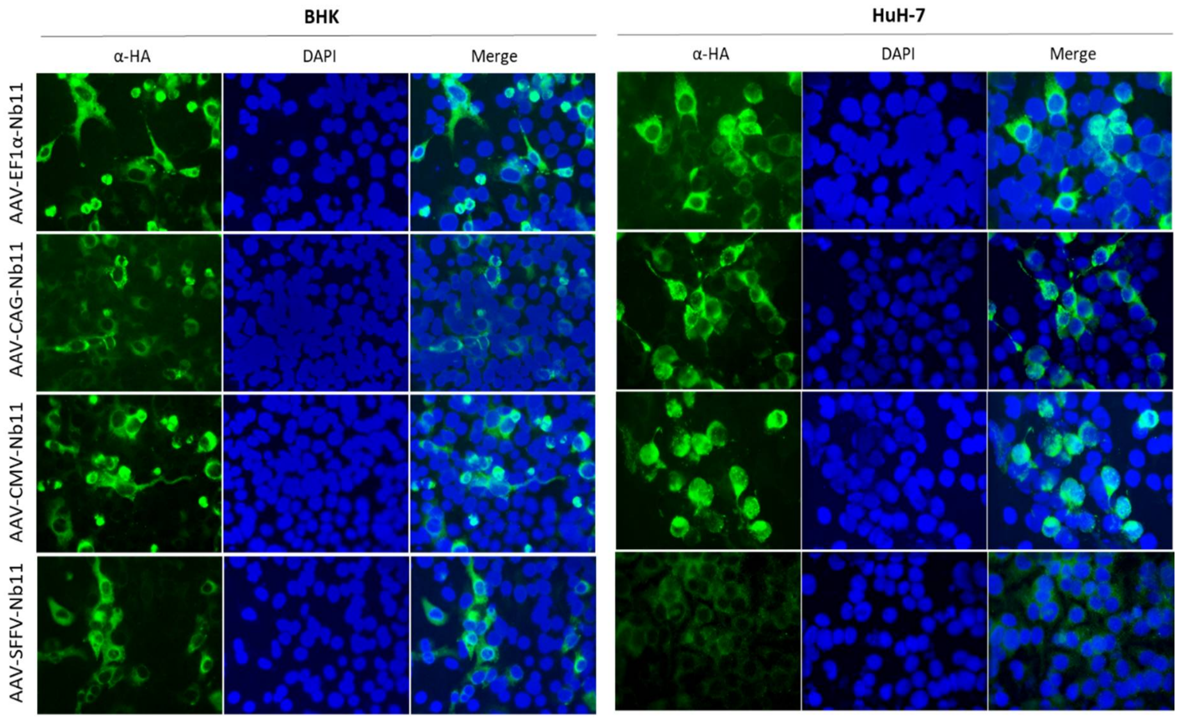

Supplementary figure 2. Immunofluorescence staining of BHK and HuH-7 cells transfected with AAV-Nb11 plasmids. 48 h post-transfection, cells were fixed with methanol and an indirect immunofluorescence was performed using an anti-HA primary antibody and a secondary antibody fused to Alexa Fluor 488. Cell nuclei were stained using mounting medium with DAPI. Cells were visualized in a Nikon Eclipse E800 microscope.

(a)

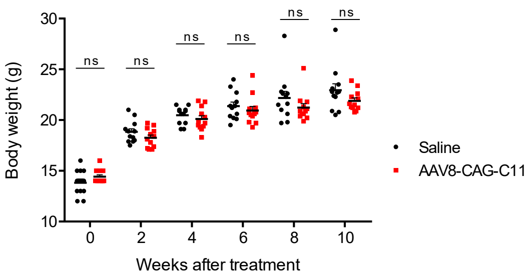

(b)

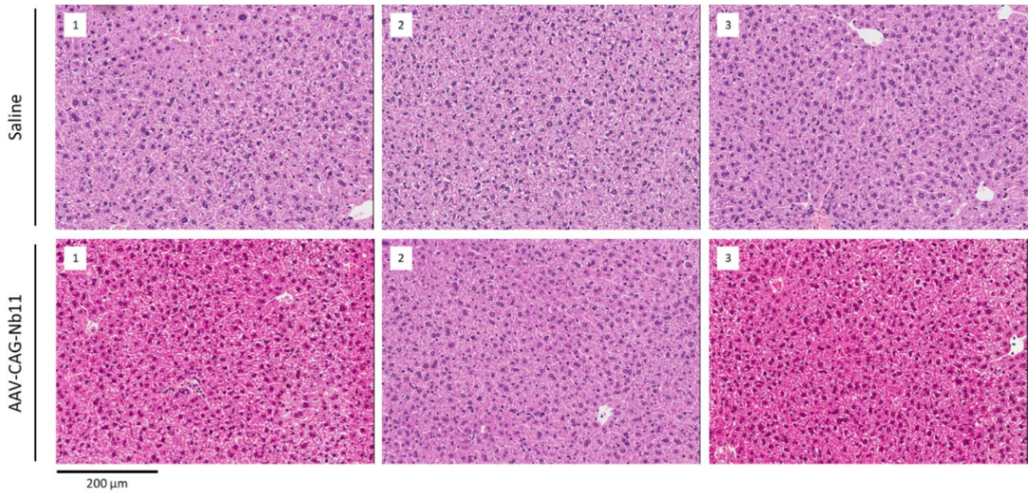

Supplementary figure 3. Evaluation of toxicity of AAV-CAG-Nb11 treatment in mice. (**a**) Body weight gain in mice that received AAV8-CAG-C11 systemically or saline. (**b**) Hematoxylin and eosin stain of liver sections of treated mice or saline. Livers were collected during animal sacrifice, from three to six weeks after tumor challenge. Each image corresponds to a different animal.
